# Tensile Behaviors of Lap-Spliced Carbon Fiber-Textile Reinforced Mortar Composites Exposed to High Temperature

**DOI:** 10.3390/ma12091512

**Published:** 2019-05-09

**Authors:** Gia Toai Truong, Sung-Ho Park, Kyoung-Kyu Choi

**Affiliations:** School of Architecture, Soongsil University, 369 Sangdo-ro, Dongjak−gu, Seoul 06978, Korea; toaigia@gmail.com (G.T.T.); cstrsh@gmail.com (S.-H.P.)

**Keywords:** tensile test, carbon fibers, alumina cement, textile reinforced mortar, high temperature, lap splice, surface treatment, prediction model

## Abstract

The tensile behaviors of textile-reinforced mortar (TRM) composites made with carbon fiber textile and alumina cement-based mortar were investigated through direct tensile tests. Three different surface treatment details in the lap splice area were used to improve the tensile behaviors of the TRM composites: carbon fiber textile impregnated by epoxy, carbon fiber textile coated with aluminum oxide powder following epoxy impregnation, and carbon fiber textile coated with aluminum oxide powder following both carbon fiber fabric attachment and epoxy impregnation. Three different lap splice lengths were used 180, 200, and 220 mm. In addition, the tensile properties of TRM composites following exposure to high temperature were investigated as well. In this test, TRM test specimens were exposed to two different temperature histories with maximum values of 250 and 350 °C. The results of the test specimens according to the test parameters were analyzed in terms of initial stiffness, cracking strength, corresponding strain at cracking, modulus of elasticity in the cracked stage, peak strength, and ultimate strain. The influence of lap splice length on the tensile behaviors of the TRM composites was analyzed and discussed. The surface treatment in the overlapping region showed ductile behavior and resulted in a significant improvement of the peak strength and ultimate strain over the untreated lap splice textile. Following exposure to high temperature, the TRM composites showed a reduction of tensile responses compared to those cured at room temperature. In addition, a prediction model developed in the previous study was used to predict the tensile behaviors of the lap-spliced carbon fiber-textile reinforced mortar composites exposed to high temperature, and the prediction by the model showed a good agreement with the experimental results.

## 1. Introduction

Various retrofitting techniques, such as steel plates, fiber sheets or plates, and composite materials including fiber-reinforced polymer (FRP), have been developed for application into strengthening and repairing existing concrete structures showing strength shortages, which are mainly due to deterioration caused by time and increasing applied loads [1,2,3,4]. Among these retrofit materials, fiber-reinforced polymer composites have been widely employed due to their ease of installation in various shapes, lightweight nature, high corrosion resistance capacity, and high tensile strength and stiffness [5,6]. In the fabrication of FRPs, organic matrices including thermoset and thermoplastic types have been used as bonding materials. However, according to the studies by Escrig et al. [7] and D’Antino and Papanicolaou [8], some drawbacks could be observed when using organic matrices in FRP composites, such as the reduction of tensile strength at high temperatures, low fire resistance, low vapor permeating capacity, and poor compatibility with substrate materials (e.g., concrete and masonry). In order to reduce such shortcomings, inorganic materials (e.g., cement-based mortars and lime-based mortars) were introduced as matrices of composite materials. In contrast to organic matrices, which were commonly combined with fiber fabrics or sheets, inorganic matrices were combined with fiber meshes or textiles. This means that inorganic matrix composite specimens consisted of fibers in the form of textiles and cement-or lime-based mortars, and they were usually named fiber (or fabric) reinforced cementitious matrix (FRCM) or textile-reinforced mortar (TRM). The use of inorganic matrices could result in improved resistance to high temperature, compatibility with substrate materials, and vapor permeability [9]. In addition, Ombres [10] and Awani et al. [11] indicated that the fiber textiles with mesh openings could provide better interlock performance and thus substantially restrict the potential debonding between fibers and mortar matrix in TRM composites. Such effectiveness when employing textile reinforcements was based on properly controlling the fiber textiles assembly as well as the casting and curing of mortar matrix [12]. 

The development of TRM systems resulted in an alternative solution to FRP composites for the repair, rehabilitation, and retrofitting of structures. Nevertheless, as the mechanical properties of TRM composites are relatively complex, better understanding is necessary for further application of the TRM composites. Many tensile tests have recently been carried out to experimentally investigate the tensile behaviors of TRM composites [13,14,15,16,17,18]. Messori et al. [19] investigated experimentally the effect of epoxy impregnation on the tensile behaviors of TRM composite specimens. The test results obtained indicated that fiber fabric impregnated by epoxy could result in enhanced interfacial bonding between fabric and matrix, and could consequently significantly increase the ultimate tensile strength and strain of the TRM composites. In studies by Signorini et al. [20] and Donnini et al. [21], the same behavior was also observed when the textile fabrics were coated with silica (SiO_2_) nano and sand following epoxy impregnation, respectively. In addition, Donnini et al. [21] also investigated whether the epoxy impregnation and sand coating might change the failure mode of the FRCM composites from fiber slippage within the matrix to fiber rupture. As a result, the use of coated fibers increased the adhesive performance at the interface between mortar and fibers and thus resulted in fiber rupture. In addition, using a higher mortar compressive strength resulted in the higher tensile strength of the FRCM composites. Additionally, Du et al. [22] demonstrated that increasing the number of textile layers significantly enhanced the tensile performance of basalt textile-reinforced concrete (TRC), and resulted in finer cracking patterns due to the effect of strain-hardening after cracking. Mesticou et al. [23], Nguyen et al. [24], and Kong et al. [25] also investigated whether the tensile behaviors of the TRM composites could be considerably affected by the use of different textile configurations, loading speed, exposure to high temperature, and curing conditions. Especially, Raoof and Bournas [26] found that the TRM could provide better bonding performance on concrete surfaces than FRP even though subjected to high temperature. Thus, at a high temperature level, the TRM exhibited excellent performance as a strengthening material by increasing shear and flexural capacities of the concrete members subjected to high temperature compared to those using FRP jacketing [27,28].

This study experimentally investigated the characteristics of TRM composite specimens through direct tensile testing. The TRM test specimens were composed of carbon fiber textile and alumina cement-based mortar, and each one had a lap splice region in the middle. The main test parameters are the lap splice length, the surface treatment details in the lap splice region, and the high temperature exposure of the test specimens. The results of the test specimens were presented and discussed in terms of failure mode and stress–strain relationships with mechanical parameters such as initial stiffness, cracking strength, corresponding strain at cracking, modulus of elasticity in the cracked stage, peak strength, and ultimate strain. Moreover, an analytical model was used to predict the mechanical properties of the lap-spliced carbon fiber-textile reinforced mortar composites exposed to high temperature.

## 2. Experimental Program

### 2.1. Materials

**Figure 1:**
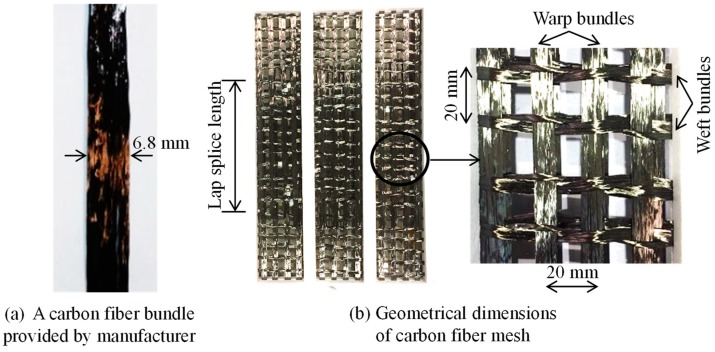
Geometrical properties of carbon fiber mesh used as textile reinforcement.

The TRM composites tested in this study were constituted of carbon fiber mesh embedded within aluminum cement-based mortar. The carbon fiber mesh, which was used as textile reinforcement, was made from carbon fiber bundles. Figure 1a shows a typical carbon fiber bundle provided by the manufacturer (Sebangfiber, Sejong, Korea). A fiber bundle has an average width and thickness of 6.8 and 0.21 mm, respectively, and consists of 24,000 filaments each with a diameter of 7 μm. The tensile strength and elastic modulus of the carbon fiber filaments are 4900 MPa and 230 GPa, respectively.

The geometrical properties of textile reinforcement used in this study are presented in Figure 1b. Note that the textile reinforcement was fabricated in the laboratory, and has the same details as that presented in the study by Kim et al. [29]. As shown in Figure 1b, the textile reinforcement has the constant space of grid (from mid-bundle to mid-bundle) of 20 mm in both directions. However, the cross-section areas of the bundles in the orthogonal directions are not equal to each other. In the warp direction, each warp bundle of the textile consisted of three carbon fiber bundles with a total thickness of approximately 0.63 mm and 72,000 carbon fiber filaments. They were kept straight while the test specimens were made so as to ensure that the tensile stresses could contribute to the carbon fiber bundles during the loading process. Meanwhile, in the weft direction, the weft bundle was in a braided shape and comprised of two carbon fiber bundles with a theoretical thickness of 0.42 mm and 42,000 carbon fiber filaments.

In this study, the lap splice lengths of the textile reinforcements were, in order, 180, 200, and 220 mm. The lap splice was also fabricated in the laboratory. The development of 200 mm lap splice length of textile reinforcements was higher than the minimum requirement (51 mm) specified in AC434 [30] and was based on the previous test results [29]. Two separated carbon fiber meshes were set to overlap each other to a length as required. As a result, within the central area of the test specimen, the number of carbon fiber filaments of each warp bundle of the textile was approximately 144,000.

The matrix used in this study was an alumina cement-based mortar, which has excellent heat resistance and fluidity. In the mortar proportion, granular sand with a granulation ratio of 2.6 was used. The ratio between water and granular sand was 1:2, and that between granular sand and fine aggregate was 1:3. The compressive strength of the mortar was tested according to Korean test standard KS L 5105 [31]. On the testing day for TRM specimens (28th day), the average compressive strength of the mortar was 51.1 MPa.

### 2.2. Test Specimens

**Figure 2:**
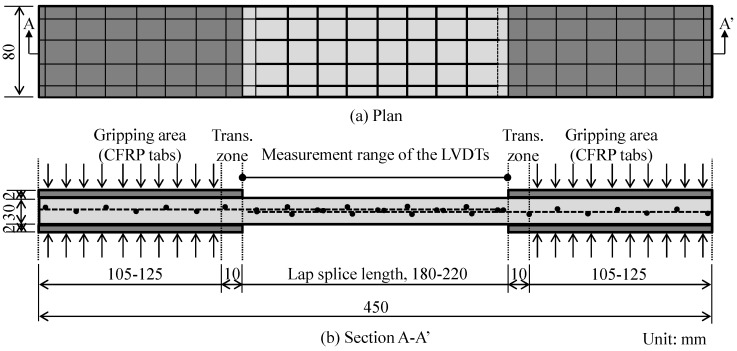
Details of test specimens.

In this study, a total of 27 TRM test specimens were made and tested under direct tensile load in conformity with ACI 549.4R-13 [32] and AC434 [30]. The details of the TRM test specimens are presented in Figure 2 and Table 1. All TRM test specimens were rectangular prisms with a length of 450 mm, a width of 80 mm, and a thickness of 30 mm (see Figure 2). The length of the TRM test specimens included the gripping areas, transition zones, and a central area for measuring the strain of the specimen. The length of the gripping area was in a range from 105–125 mm, and that of the central area was in a range from 180–220 mm, while the length of the transition zone was constant at 10 mm. The variation of the length of the central area was determined to be the same as the lap splice length of textile reinforcement located within the central part of the test specimen. In this research, as mentioned above, the lap splice lengths of the textile reinforcements were 180, 200, and 220 mm. At the gripping areas (at both ends of the test specimen), 2 mm thick carbon fiber reinforced polymer (CFRP) tabs were used as gripping materials in order to strengthen the specimen ends so as to avoid premature failure of the mortar matrix within or near the clamping area. Such premature failure of the mortar matrix was attributed to the use of clamping grips inducing the compressive stress concentration to the specimen ends [31]. Moreover, according to Kim et al. [29], in some cases, the cracks could occur in the transition zone, which was also influenced by the clamping pressure. Consequently, the CFRP tabs in the gripping areas were lengthened to the transition zones so as to avoid such cracks (see Figure 2). Regards to the thickness of the test specimens, in general, thin layer of TRM materials was used [16,20,21] to reduce the weight, to possibly approach a composite-like behavior, and to limit the lever arm of the reinforcing fibers with respect to the surface of the substrate. However, in this study, to use the TRM materials in high-temperature levels, a thick layer of the TRM materials was used. In addition, the thickness of the TRM specimens in this study was matched to the requirement mentioned in the study by De Santis et al. [12].

Three different test series corresponding to three main test parameters were developed and experimented in this study, including lap splice length, surface treatment details in the lap splice area, and maximum temperature when exposed to high temperature (see Table 1). Note that on the second day after casting, all test specimens were demolded then cured in the laboratory condition with a temperature of 11 ± 2 °C and a relative humidity of 95%. 

**Figure 3:**
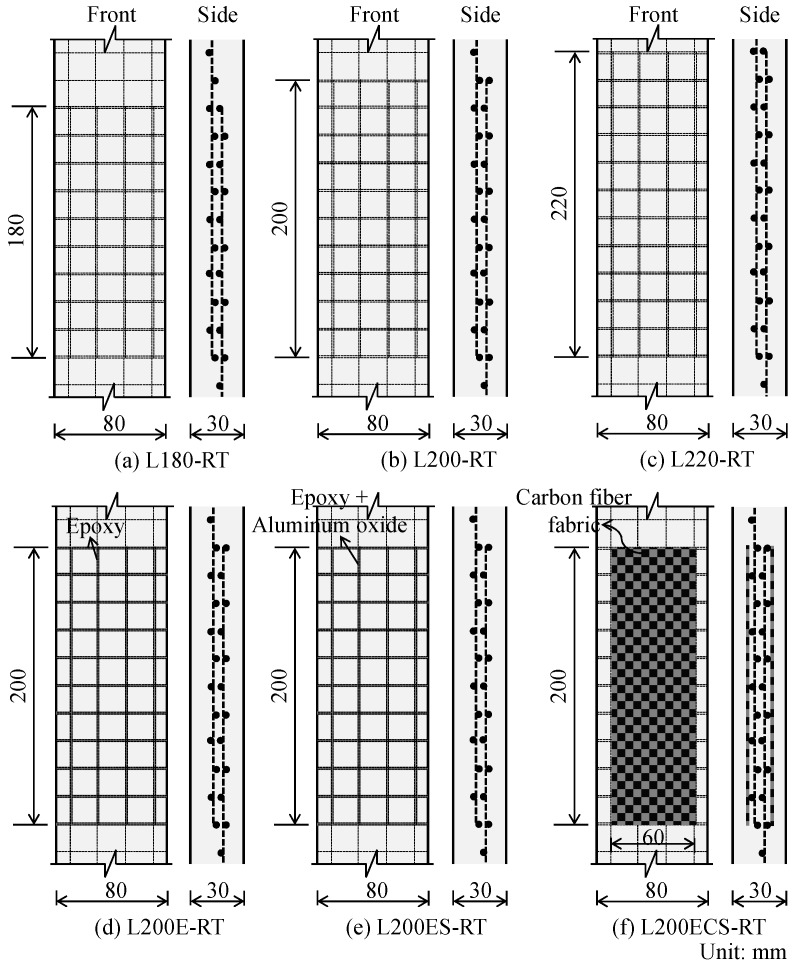
Details in lap splice area.

In the case of the first test series, in order to investigate the effect of textile lap splice length on the tensile behaviors of TRM test specimens, three different lap splice lengths of the textile reinforcement were used: 180, 200, and 220 mm. The details of lap splice length are presented in Figure 3a–c. For each parameter, two replicas of test specimens were made and tested without exposure to high temperature (or at room temperature) (see Table 1). In Table 1, the specimen name indicates the lap splice length and the testing condition. For example, specimen L180-RT means that the lap splice length of the test specimen was 180 mm and it was exposed to room temperature prior to testing. 

In the second test series, three different surface treatment details in lap splice joint were developed and experimented in this study. The purpose of this was to improve the bonding performance between fibers and mortar matrix. For each method, three replicas of the test specimens were made and tested. The details for lap splice joint in this study are presented in Figure 3d–f. In the figure, all of the test specimens in the second series had the same lap splice length of 200 mm. Figure 3d describes the details of the TRM specimen named L200E-RT having a lap splice length of 200 mm and exposed to room temperature. In this test specimen, the carbon fiber filaments of the textile reinforcement mesh were fully impregnated with epoxy resin in the overlapping region with a weight of approximately 60 g. In the case of the specimen named L200ES-RT (Figure 3e), the carbon fiber filaments of textile reinforcement mesh in the overlapping region were first impregnated with epoxy resin with a weight of approximately 60 g, then the carbon textile was coated with aluminum oxide (Al_2_O_3_) powder having a diameter of 250 μm [33,34] using a spraying gun at high speed. Both sides of the carbon fiber textile were blasted so as to ensure uniform surface treatment. The use of aluminum oxide powder for surface treatment was considered in this study since it has been found to improve the bond performance between carbon fiber and adhesive materials [35]. The details of the specimen named L200ECS-RT are presented in Figure 3f. In this case, similar to the L200ES-RT specimen, carbon fibers in the overlapping region were first impregnated with epoxy resin with a weight of approximately 60 g; then, two carbon fiber fabrics having a length of 200 mm, a width of 60 mm, and a thickness of 0.21 mm were attached to clamp such carbon fiber mesh using epoxy resin. Finally, the remaining sides of carbon fiber fabrics were coated with aluminum oxide powder. The purpose of this was to convert the smooth surface of the carbon fiber fabric into a rough surface, which could result in an improvement of bonding characteristics between the carbon fiber and mortar matrix [36]. In the second series, the terms “E”, “ES”, and “ECS”, which were named following the applied details, were added in order to distinguish the TRM test specimens. 

In the third series, similar to the second series, the test specimens also had the same lap splice length of 200 mm. The details of the test specimens in this third series were nearly the same as those in the first and second series (see Figure 3). In addition, the test specimens in the third series were exposed to high temperature then cooled down prior to direct tensile testing. The maximum temperatures applied to the TRM specimens were approximately 250 and 350 °C, respectively. Thus, for the purpose of distinguishing the TRM test specimens with and without exposure to high temperature, the specimens were modified by replacing the term “RT”, meaning the room temperature, by the maximum temperatures of the specimens exposed to high temperature. For example, as presented in Table 1, specimen L200ES-250 indicates that the TRM test specimen has a lap splice length of 200 mm, the lap splice joint was impregnated with epoxy resin and coated with aluminum oxide powder, and the TRM specimen was then exposed to high temperature with a maximum temperature of 250 °C before testing. In the case of specimens exposed to a maximum temperature of 250 °C, two replicas of test specimens were made and tested. In the case of specimens exposed to a maximum temperature of 350 °C, only one replica of the test specimens was examined (Table 1). Since a few number of test specimens have been made and tested, to enhance the test results reliability, additional test specimens corresponding to each test parameter are needed in the further research.

### 2.3. Test Setup and Measurements

**Figure 4:**
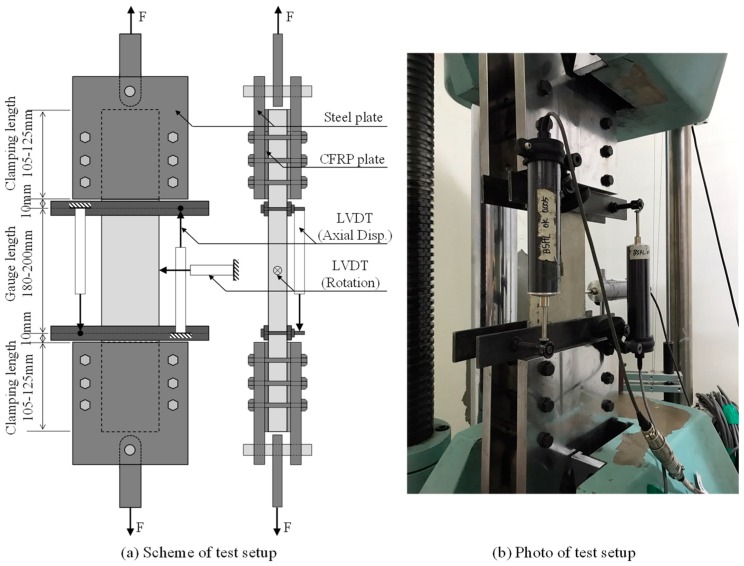
Tensile test setup.

The test setup of the tensile tests for TRM specimens is presented in Figure 4. In Figure 4a, prior to testing, the gripping areas of the TRM test specimens were clamped with a couple of steel plates, and then fastened by six bolts. This clamping method was selected based on the studies by Kim et al. [29] and Leone et al. [37]. The clamping force induced by the fastening force of these six bolts was considered to be the gripping pressure applied to the test specimens. In this study, the clamping force applied to the specimen surface was approximately 126 kN, which resulted in a maximum clamping stress of 12.6 MPa. This clamping stress was much lower than the compressive strength of the matrix (51.1 MPa), thus the crushing of the mortar could be avoided. In addition, the slippage between the CFRP and steel plates could also be prevented. The alignment of the test specimen and steel plates is very important for avoiding a bending moment that could arise during the loading. Thus, the test specimens were connected with a testing machine through a steel bar which could rotate freely (see Figure 4a). A universal testing machine (UTM, Kyoungsung Testing Machine Co., Ansan, Korea) with a loading capacity of 1000 kN was used for tensile tests in this study (Figure 4b). The tests were carried out with displacement control at a rate of 0.5 mm/min, as specified in ASTM D3039 [38].

The tensile deformation of the test specimens was measured by two linear variable displacement transformers (LVDT, Tokyo Measuring Instruments Laboratory Co., Tokyo, Japan) placed on the same side of the specimen. The LVDTs were installed along the longitudinal direction of the test specimens with a steel frame support, the span of which could be changed from 180 to 200 mm in order to match the length of the central area. In addition, another LVDT was centrally placed on the transverse direction of the test specimen in order to measure any unexpected rotation of the specimen due to load damage (see Figure 4). 

**Figure 5:**
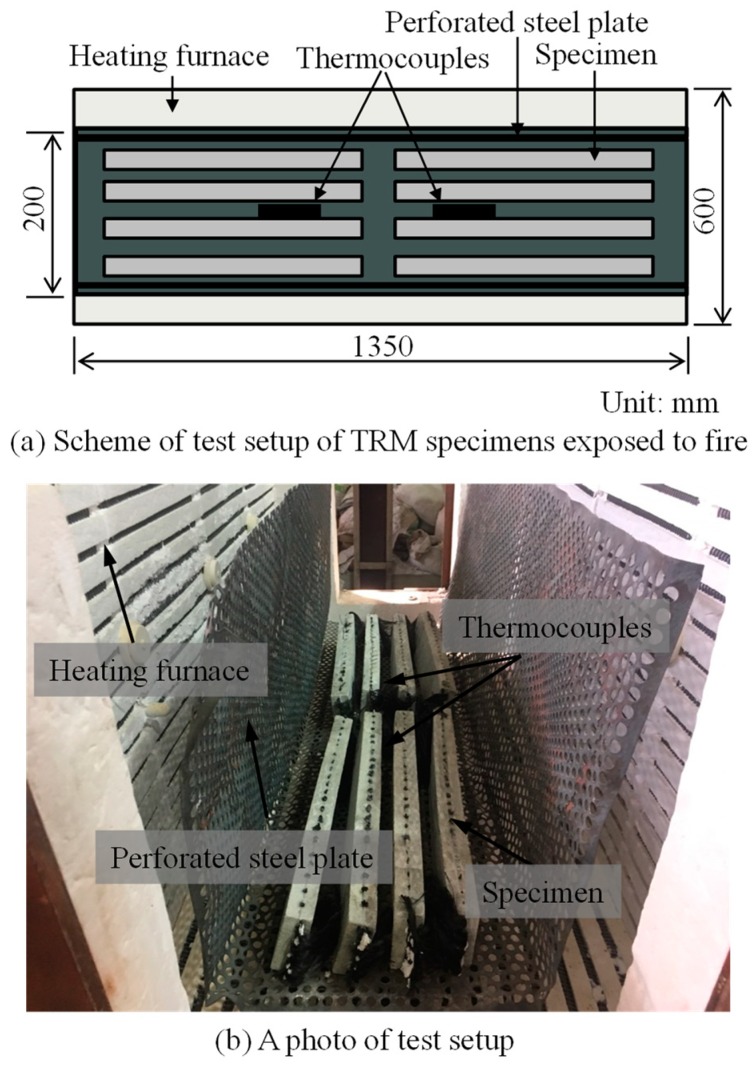
Test setup of textile-reinforced mortar (TRM) specimens exposed to fire.

In the third series, the TRM test specimens were exposed to high temperature prior to direct tensile testing. Figure 5 shows the test setup of the TRM specimens exposed to high temperature. From the figure, it can be seen that the test specimens were placed in a heating furnace and enclosed by perforated steel plates. Such perforated steel plates were used in order to prevent damage to the heating furnace from mortar breakage during firing. The temperature in the furnace was increased at a heat rate of 17.5–25 °C/min until the target temperature was attained. Two thermocouples were placed inside the heating furnace in order to measure the temperatures of the test specimens. Upon reaching the target temperature, the furnace was cooled down with a cooling rate of 2.4–2.8 °C/min. The test specimens were still kept inside the furnace during this stage so as to avoid any sudden change of temperature, which could worsen the performances of the test specimens. After 1.5 h, the test specimens were taken out and stored in the laboratory until reaching room temperature; direct tensile tests of the TRM test specimens were then executed (see Figure 4 for the test setup).

## 3. Experimental Results and Discussions

### 3.1. Temperature–Time Relationships

**Figure 6:**
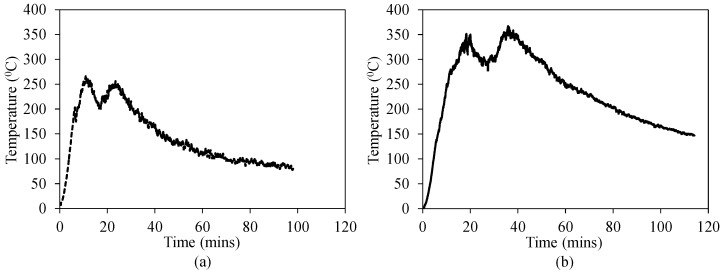
Temperature-time relationship of TRM test specimens in the third series: (**a**) maximum temperature of 250 °C and (**b**) maximum temperature of 350 °C.

Figure 6 shows the temperature versus time progress of TRM test specimens in the third series. Note that the temperature obtained was the furnace temperature. In Figure 6a, in the first heating regime, the temperature rapidly increased up to approximately 250 °C within 10 min. After that, from the 10th to 25th minute, the temperature fluctuated in a range from approximately 200 to 250 °C. Then, the heating furnace was cooled down gradually, and after 90 min, the temperature was 88.6 °C. In the second heating regime (Figure 6b), the process was similar to that of the first one. However, after the first 20 min, the maximum temperature was measured at approximately 350 °C. The fluctuation of the second temperature curve occurred from the 20th to 40th minute, and the temperature was in a range of approximately 300–350 °C. After this stage, the furnace was cooled down gradually; and after 90 min, the temperature was 181.4 °C. As mentioned above, during the cooling process, the TRM test specimens were still kept inside the furnace so as to avoid thermal shock, as the difference between the temperature inside (maximum 250–350 °C) and outside (11 ± 2 °C) the furnace was quite high, which could result in poor results of the test specimens. After 90 min, the test specimens were taken out and stored in the laboratory until reaching the room ambient temperature. Finally, direct tensile tests were performed in order to determine the mechanical characteristics of these TRM test specimens.

### 3.2. Crack Pattern and Failure Mode 

**Figure 7:**
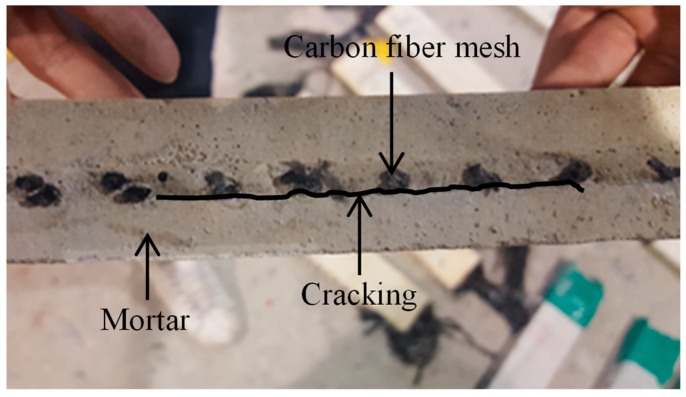
Crack pattern of a typical TRM test specimen following exposure to high temperature.

Figure 7 presents the crack pattern of a typical TRM test specimen after being exposed to high temperature. In the figure, it was found that a minor crack appeared along the longitudinal direction of the test specimen; the crack was located at the interface between carbon fiber mesh and mortar matrix. The appearance of such cracking following exposure to high temperature could reduce the interfacial bonding between carbon fiber mesh and mortar matrix, and could thus result in harmful effects on the tensile behaviors of the TRM test specimens. 

**Figure 8:**
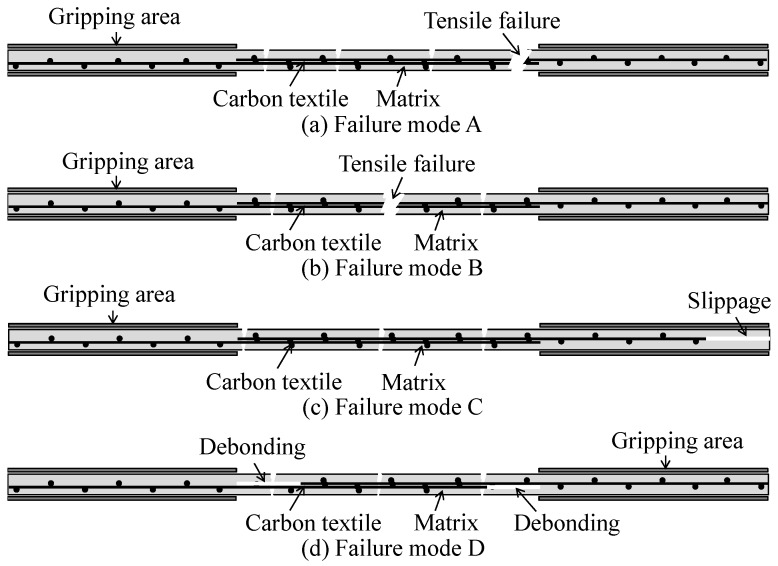
Failure modes of direct tensile tests of TRM test specimens.

The possible failure modes of TRM specimens during tensile testing are presented in Figure 8. Failure mode A (Figure 8a) indicates a failure at the transition zone near the gripping area. According to the study by Leone et al. [37], such tensile failure occurs due to the biaxial stress in the transition zone, including compressive and tensile stresses induced by the clamping grips and applied tensile force, respectively. Figure 8b describes failure mode B, which is caused by carbon fibers rupturing after cracking of the mortar matrix. Failure mode C (Figure 8c) is caused by the mortar matrix cracking and the subsequent slippage of carbon fibers within the mortar matrix at the gripping zone. Meanwhile, failure mode D (Figure 8d) involves debonding between carbon fiber textiles, regardless of the appearance of mortar matrix cracking along the length of the test specimens. 

**Figure 9:**
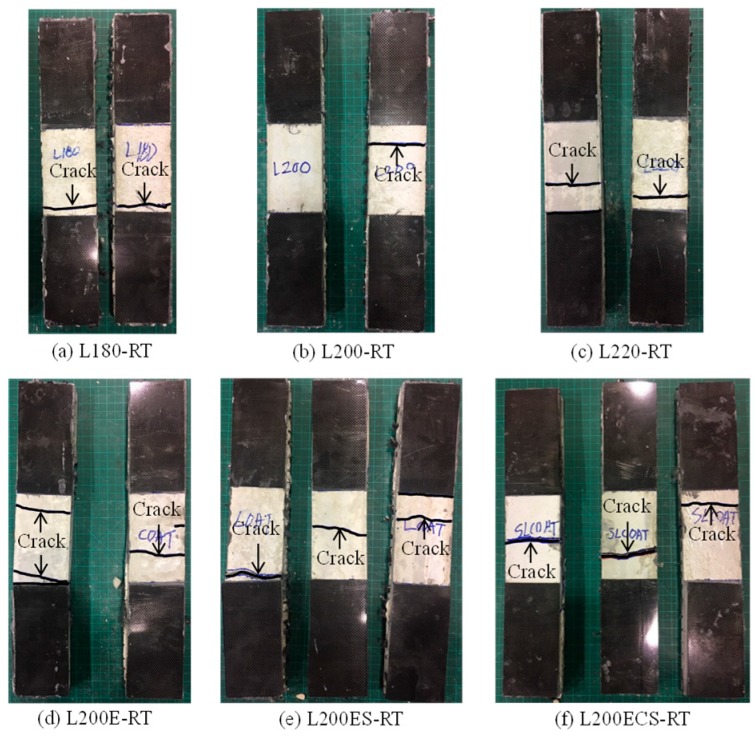
Failure modes of TRM test specimens in the first and second series.

**Figure 10:**
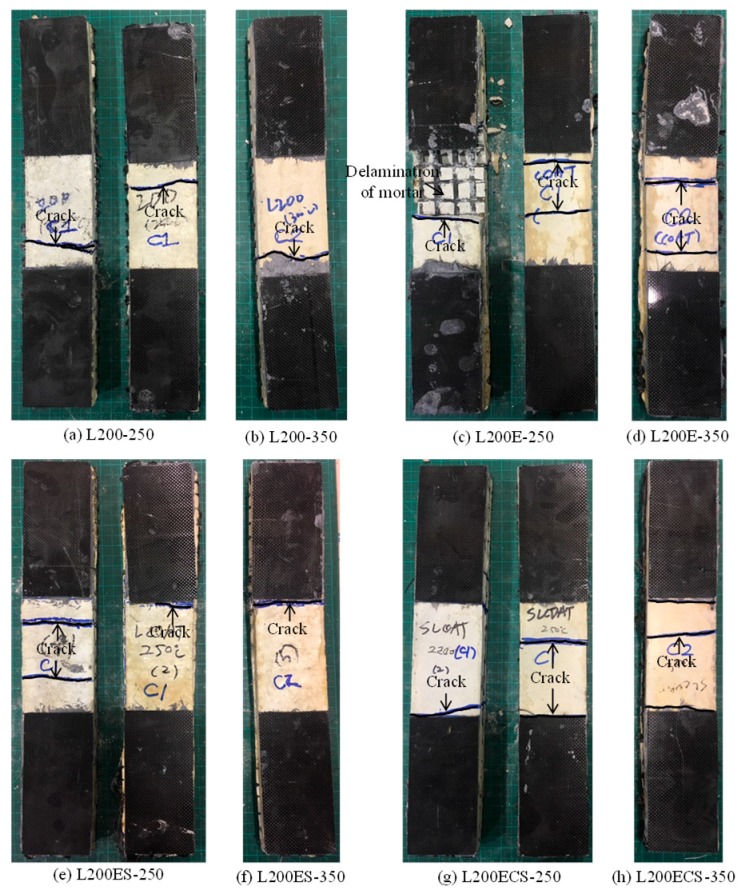
Failure modes of TRM test specimens in the third series.

The crack patterns of the TRM test specimens in this study are presented in Figure 9 and Figure 10. In general, the failure modes obtained from the test results of the test specimens were denoted as A or B (see Table 2). The slippage of carbon fibers within the gripping zone (failure mode C) and debonding between fiber textiles (failure modes D) were not observed. In addition, the figures also show that the crack patterns of the TRM test specimens involved only one or two cracks. This means that following the formation of the first mortar matrix crack, the axial force was immediately transferred to the carbon fiber textile. At this stage, the tensile strength and strain of the TRM specimens would be mainly governed by textile reinforcement. 

In the case of the L180-RT specimen (Figure 9a) using a lap splice length of 180 mm and cured at room temperature, the warp carbon fibers were ruptured after the first crack of the mortar matrix. However, L180-RT finally failed by mode A due to the crack development near the gripping area. 

The L200-RT specimen (Figure 9b) using a lap splice length of 200 mm and cured at room temperature showed failure mode of A. In the cases of the specimens L200-250 (Figure 10a) and L200-350 (Figure 10b), which were exposed to high temperature, the failure mode was the same as that of L200-RT. A similar failure mode was also observed in the test specimens L200E-350 (Figure 10d) and L200ES-350 (Figure 10f). In the case of the L220-RT specimen (Figure 9c), the crack of the mortar matrix occurred first; subsequently, the warp carbon fibers were ruptured. However, as shown in Figure 9c, such cracks occurred both near the gripping area and in the central zone, the failure mode of L220-RT specimen could be classified into A or B. Other test specimens showed the failure mode of A or B, like the L220-RT specimen. Separately, in the case of specimen L200E-250, because the crack along the loading direction occurred during the exposure to high temperature, the mortar matrix was delaminated after cracking induced by unidirectional tension (see Figure 10c). Regarding specimen L200ECS-350, as shown in Figure 10h, cracks generally occurred both in the center of the specimen and in the vicinity of its ends, but the final failure mode was denoted as B due to the rupturing of warp carbon fibers in the central zone. 

In general, based on the test results, the cracking patterns of the TRM test specimens did not appear to be significantly affected by exposure to high temperature.

### 3.3. Idealized Stress–Strain Curves of Textile-Reinforced Mortar (TRM) Test Specimens 

**Figure 11:**
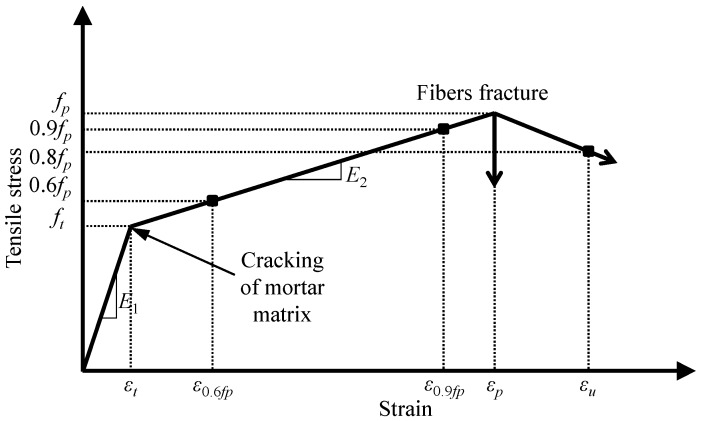
Idealized stress–strain curve of TRM test specimens.

Figure 11 shows an idealized stress–strain curve of the TRM test specimens examined in this study. In Figure 11, the curve was assumed as a linearized model including un-cracked and cracked stages, which was distinguished by the transition point (or cracking point), for simple investigation. The first linear segment represents the un-cracked stage and is characterized by the elastic modulus $E_{1}$ (or initial stiffness). The first stage is governed by the mechanical properties of the mortar matrix and carbon fiber meshes based on their good interfacial bonding. The second segment corresponds to the cracked stage of the TRM specimens and is characterized by the modulus of elasticity of $E_{2}$. In the second stage, it is mainly governed by the mechanical properties of carbon fiber meshes, because the mortar matrix was cracked and thus did not substantially contribute to sustaining the applied load. Following this, the specimens showed a sudden or gradual strength reduction. In fact, in the specimens with surface treatment and/or those exposed to high temperature, the stress–strain relationship showed fluctuation and non-linear behaviors after cracking. However, in this study, for consistent comparison across all specimens, the same mechanical parameters ($E_{1}$, $E_{2}$, $f_{t}$, $f_{p}$, $\varepsilon_{t}$, and $\varepsilon_{u}$) based on the linear model presented in Figure 11 were used. 

In this study, the mechanical properties of TRM test specimens undergoing direct tensile loading were investigated in terms of elastic moduli $E_{1}$ and $E_{2}$, which correspond to the slopes of the un-cracked and cracked stages of the specimens, respectively, as well as cracking strength ($f_{t}$), cracking strain ($\varepsilon_{t}$), peak strength ($f_{p}$), and ultimate strain ($\varepsilon_{u}$). The first elastic modulus $E_{1}$ (or initial stiffness) was determined in the range of the cracking strength and cracking strain. In contrast, the elastic modulus of cracked stage can be calculated as Equation (1) according to ACI 549.4R-13 [31].

(1) $$E_{2}=\Delta f/\Delta \varepsilon =\left(0.9f_{p}-0.6f_{p}\right)/\left(\varepsilon_{0.9f_{p}}-\varepsilon_{0.6f_{p}}\right)$$

**Figure 12:**
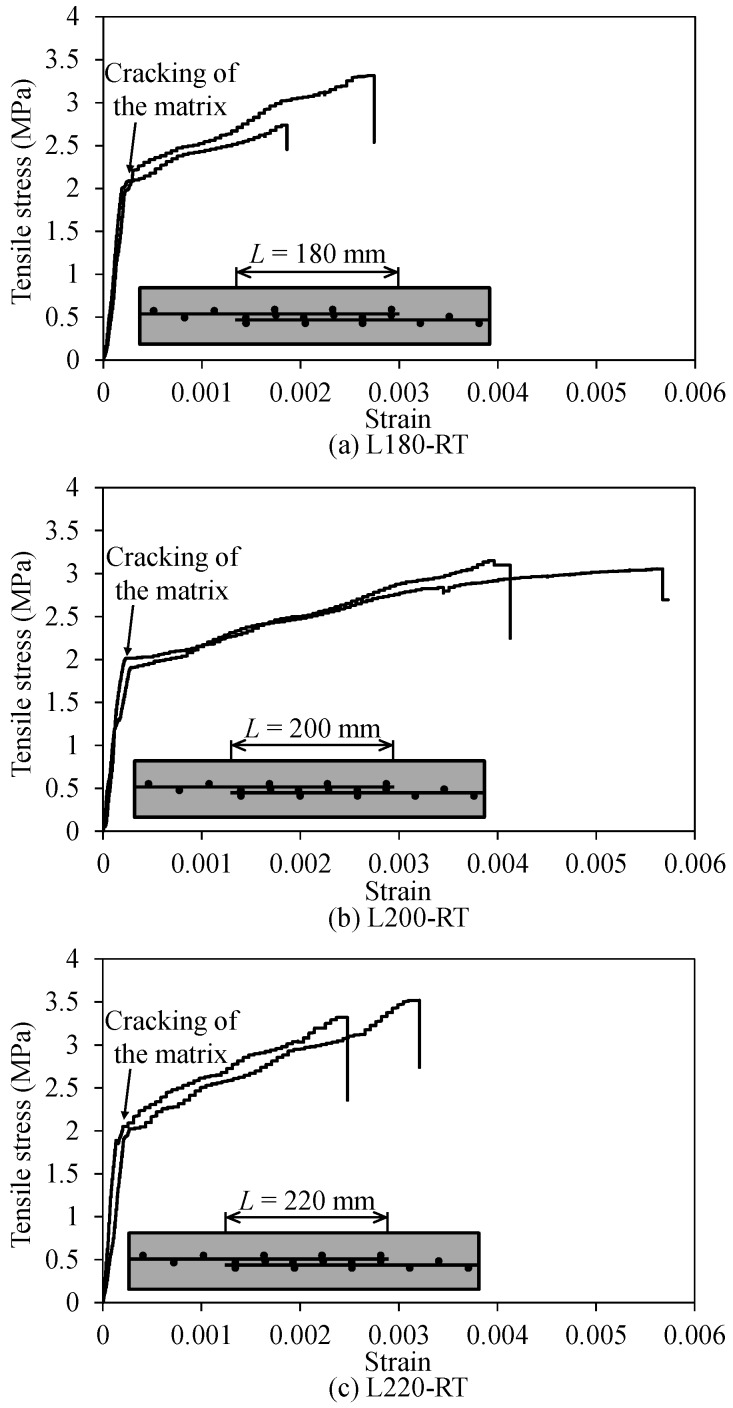
Stress–strain relationship of test specimens in the first series.

However, in some cases, the strength value of $0.6f_{p}$ was less than the cracking strength ($f_{t}$) due to local fluctuation of the stress–strain curves, thus the elastic modulus of cracked stage would be calculated using the cracking strength and cracking strain rather than the values of $0.6f_{p}$ and $\varepsilon_{0.6f_{p}}$. In addition, the tensile stress of the test specimens was calculated as the applied load divided by the gross cross-sectional area of the specimens [39]. The ultimate strain ($\varepsilon_{u}$) was defined as the point in which the applied load was equal to approximately 80% of the peak load in the descending branch. However, in the case of the first test series (Figure 12), after reaching the peak load, a sudden drop of the applied load was observed; thus, the ultimate strain ($\varepsilon_{u}$) was determined to be equal to the strain at the peak load. Meanwhile, in the second and third series (Figures 14 and 16), upon reaching the peak load, a gradual degradation of the applied load was observed in the descending branch. Thus, the ultimate strain ($\varepsilon_{u}$) could be determined in the descending branch; however, when the test was terminated and no data could be obtained up to 80% of the peak load in the descending branch, the ultimate strain was defined at the final failure point of the test specimens. The test results of the TRM specimens in this study are listed in Table 2. In the table, the coefficients of variation (COV) of each parameter were also provided in order to estimate the variability of the test results. 

### 3.4. Effect of Lap Splice Length on Tensile Behavior of TRM Test Specimens

Figure 12 presents the stress–strain response curves of the TRM test specimens in the first series. From the test results, it can be seen that the specimens in the first series showed a similar behavior to the idealized curves presented in Figure 11. In the un-cracked stage, the stress–strain curves of the TRM test specimens exhibited linearly with a high slope (or initial stiffness), which was due to the evident contribution of the mortar matrix, until the cracks occurred within the mortar matrix. After cracking of the mortar matrix, no load drop was observed in the experimental curves. In the cracked stage, the test specimens in the first series also showed linear stress–strain curves until the fracturing of carbon fibers, which induced a sudden drop at the ends of the experimental curves (Figure 12). However, the slopes of the curves in this stage were relatively low compared to those in the un-cracked stage. This is understandable, because the contribution of the mortar matrix to the tension stiffening of the TRM test specimens was significantly reduced following the cracking of the mortar matrix.

**Figure 13:**
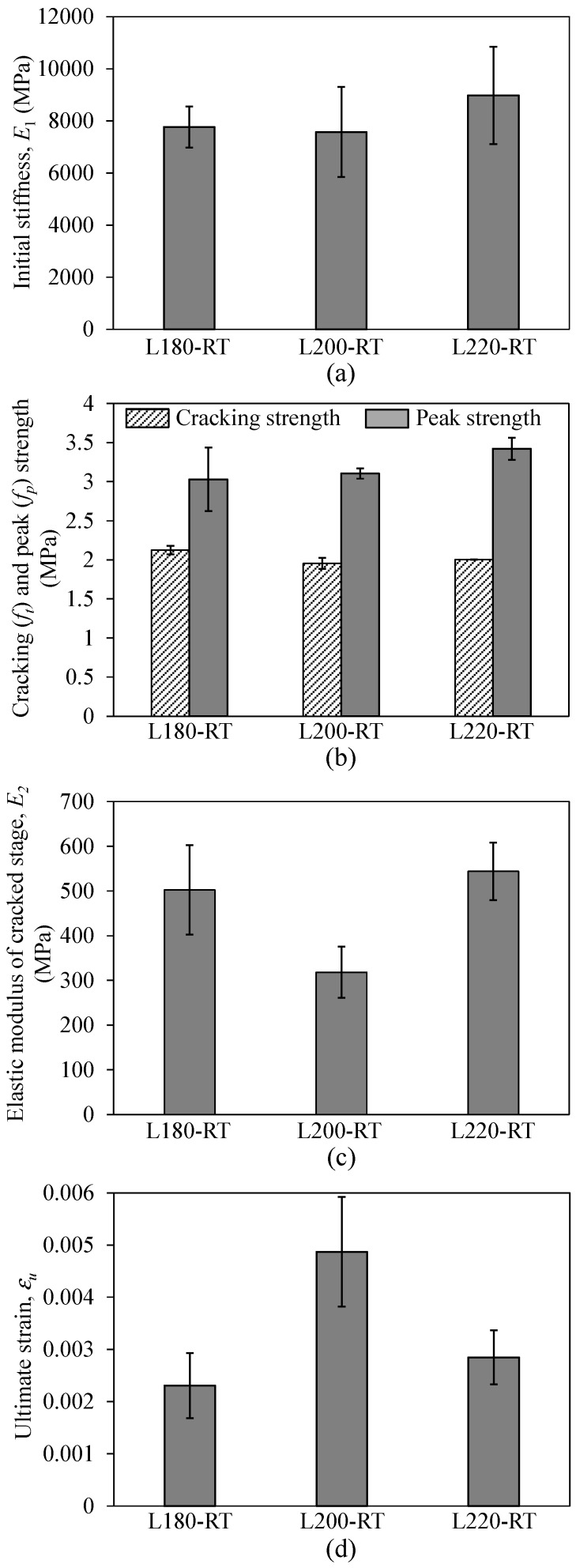
Effect of lap splice length on (**a**) initial stiffness, (**b**) cracking and peak strengths, (**c**) elastic modulus of cracked stage, and (**d**) ultimate strain of TRM specimens.

Figure 13 illustrates the effect of lap splice length on the tensile behaviors of the TRM test specimens. In Figure 13a, the initial stiffnesses of the TRM specimens were nearly the same. The average initial stiffness of the L180-RT specimen was 7770.8 MPa with a coefficient of variation (COV) of 0.102, and those of L200-RT and L220-RT were 7573.0 MPa with a COV of 0.229 and 8983.4 MPa with a COV of 0.208, respectively (see Table 2). Similarly, the use of various lap splice lengths did not affect the cracking strengths of the TRM specimens, which were almost the same with values ranging from 1.95 to 2.12 MPa (see Figure 13b and Table 2). Meanwhile, as shown in Figure 13b, it is clear that increased lap splice length could result in a slight increase of the peak strength. The use of the 200 and 220 mm lap splice lengths in the L200-RT and L220-RT specimens increased the peak strengths up to 2.5 and 12.9%, respectively, compared to that of L180-RT. This was attributed to the fact that the fabrication of the overlapping joint led to textile reinforcement with two layers in the central zone of the TRM test specimens. The use of a higher lap splice length could result in a higher tensile strength [22]. In contrast, the TRM specimens exhibited fluctuation in the elastic modulus of the cracked stage ($E_{2}$) when a long lap splice length was used (Figure 13c). The 200 mm lap splice length showed less elastic modulus in the cracked stage but higher ultimate strain in comparison with the other cases (Figure 13d). Thus, further investigation may be needed in order to understand the ultimate strain and the elastic modulus of cracked stage.

### 3.5. Effect of the Surface Treatment Details of Lap Splice on Tensile Behavior of TRM Test Specimens 

**Figure 14:**
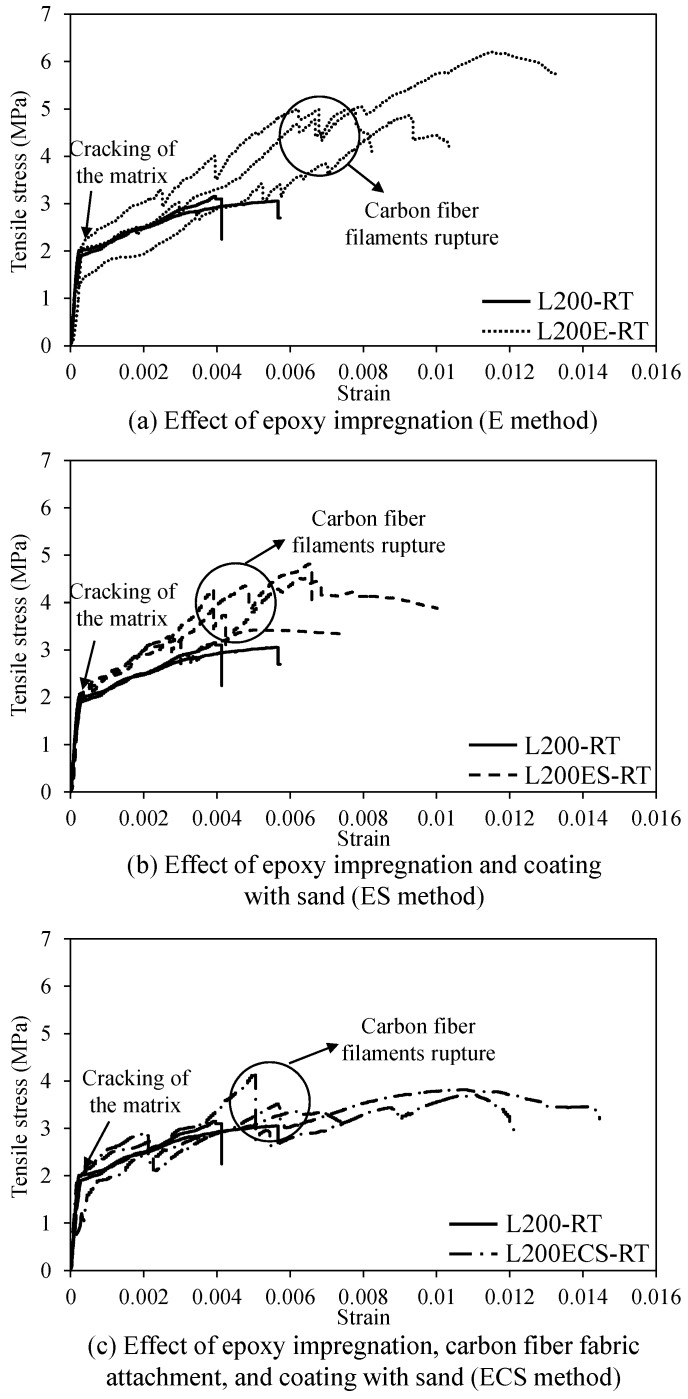
Stress–strain relationship of test specimens in the second series.

Figure 14 presents the stress–strain relationship of TRM specimens in the second series. In general, the specimens in the second series showed fluctuation and non-linear behaviors of the stress–strain curves. However, despite showing fluctuation, the specimens in the second series demonstrated similar behavior with un-cracked and cracked stages, as shown in Figure 11. The stress–strain curves of the TRM test specimens correspond to linear elastic with a high slope and ended with the formation of the first crack in the mortar matrix. Following the cracking of the matrix, no significant load drop was observed in the experimental curves of the TRM specimens. In the cracked stage, no further cracks appeared and the existing cracks continued to widen, leading to increased longitudinal deformation of the textile reinforcement. With continuing to apply load, several carbon fiber filaments were first ruptured, and subsequently, the stress–strain curves of the TRM specimens were in a non-linear response with fluctuation, as shown in Figure 14. The final failure of the TRM test specimens in the second series occurred with the ultimate tensile strain ($\varepsilon_{u}$) ranging from 0.0081 to 0.0113 (see Table 2), which was substantially higher than the ultimate tensile strain of L200-RT. Note that the failure of the test specimens in the second series occurred gradually after reaching the peak load. The other mechanical parameters obtained from the test results of the specimens in the second series are also presented in Table 2. 

**Figure 15:**
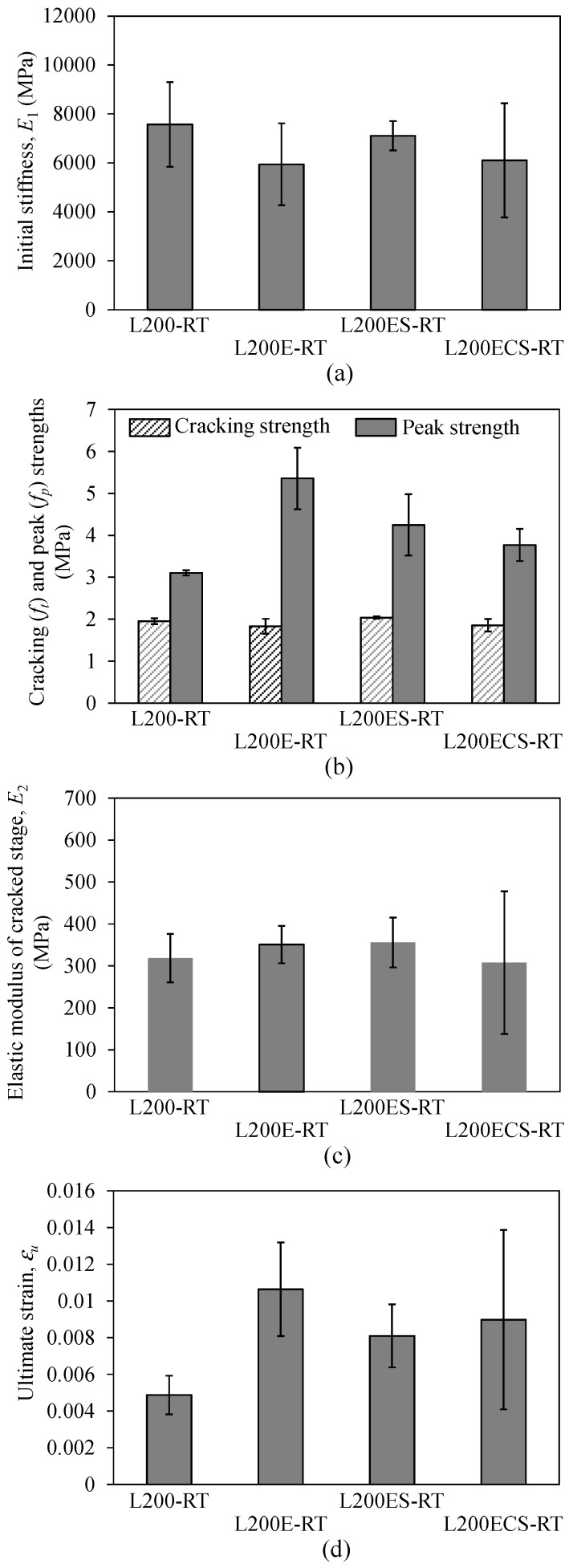
Effect of the details in lap splice area on (**a**) initial stiffness, (**b**) cracking and peak strengths, (**c**) elastic modulus of cracked stage, and (**d**) ultimate strain of TRM specimens.

Comparison between the TRM specimens with and without surface treatment details in the lap splice area is also presented in Figure 15. As shown in Figure 15a–c, in general, the initial stiffnesses ($E_{1}$), cracking strengths ($f_{t}$), and elastic moduli of cracked stage ($E_{2}$) of the L200E-RT, L200ES-RT, and L200ECS-RT specimens were almost the same as those of control specimen L200-RT. This means that the use of the surface treatment details in the overlapping region did not result in considerable differences of the initial stiffness, cracking strength, or stiffness following the cracking of mortar. Separately, as presented in Figure 15c and Table 2, the L200ECS-RT specimen exhibited a large scatter of elastic modulus of cracked stage with a coefficient of variation (COV) of 0.653. Such a large scatter in the deformation measurement of the L200ECS-RT specimen was attributed to the non-homogenous stress distribution among the bundles as well as the contribution of the mortar matrix to the tension stiffness of the specimens following the cracking of mortar [40]. 

In Figure 15b,d, it can be seen that the use of the surface treatment details in the overlapping region resulted in improvements in peak strength ($f_{u}$) and ultimate strain ($\varepsilon_{u}$), respectively. These were attributed to the fact that the use of carbon fiber textile impregnated by epoxy, coated with aluminum oxide powder, or covered with carbon fiber fabric layers within the overlapping region was able to produce an enhancement in the bonding characteristic at the interface between fibers and mortar matrix. In terms of peak strength (Figure 15b), the L200E-RT, L200ES-RT, and L200ECS-RT specimens exhibited significant increases of approximately 72.5, 36.8, and 21.4%, respectively, compared to that of the L200-RT specimen. Figure 15b also showed that the specimen L200E-RT impregnated by epoxy exhibited more effectiveness in the increase of the peak strength than the remaining details. The ratio ($f_{p}/f_{t}$) between the peak strength and cracking strength of L200E-RT was relatively high at 2.93; those of L200ES-RT and L200ECS-RT were 2.08 and 2.03, respectively. This indicates that in the case of specimen L200E-RT impregnated by epoxy, the increased peak strength after the cracking of mortar matrix was higher than the other details. In the case of ultimate strain shown in Figure 15d, significant increases were achieved for all test specimens using the surface treatment details in the lap splice area. The L200E-RT, L200ES-RT, and L200ECS-RT specimens exhibited increases of 116.3, 65.3, and 83.7%, respectively, compared to that of the L200-RT specimen. In addition, as shown in Figure 15d, similar to the peak strength, the use of carbon fiber reinforcement impregnated by epoxy only could result in more effectiveness in increase of the ultimate strain than the other details. 

Obviously, as mentioned above, the use of surface treatment improved significantly the tensile behaviors of TRM specimens as investigated in the previous studies [19,20,21]. However, the use of carbon fiber reinforcement impregnated by epoxy only might be the most effective than the other details including carbon fiber textile coated with aluminum oxide powder following epoxy impregnation, and carbon fiber textile coated with aluminum oxide powder following both carbon fiber fabric attachment and epoxy impregnation. This is due to the non-uniform bonded surface between bundles and mortar [21]. In the case of L200E-RT specimens, coating with epoxy only guaranteed a uniform bonded surface of the bundles (or more smooth), and the mortar is able to penetrate inside the bundles, which play a role as a stiffening core. When using carbon fiber textile coated with aluminum oxide powder following epoxy impregnation in L200ES-RT specimens, the surface of bundles become roughness. However, the fact unintentionally limited the penetration of mortar inside the bundles, and thus partially limited the stress-transfer mechanism between mortar and textile. Similar observations could be found in the case of L200ECS specimens using carbon fiber textile coated with aluminum oxide powder following both carbon fiber fabric attachment and epoxy impregnation.

### 3.6. Effect of Temperature on Tensile Behaviors of TRM Test Specimens 

**Figure 16:**
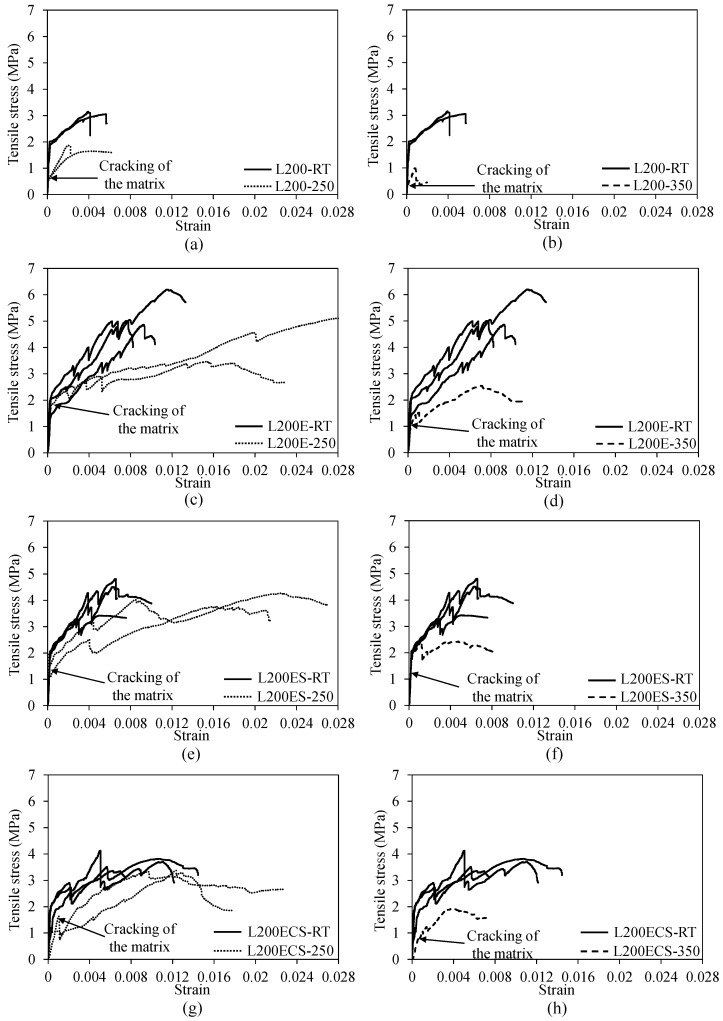
Stress–strain relationship of (**a**) L200 at 250 °C, (**b**) L200 at 350 °C, (**c**) L200E at 250 °C, (**d**) L200E at 350 °C, (**e**) L200ES at 250 °C, (**f**) L200ES at 350 °C, (**g**) L200ECS at 250 °C, and (**h**) L200ECS at 350 °C.

The stress–strain response curves and mechanical parameters of the TRM test specimens in the third series are presented in Figure 16 and Table 2, respectively. Note that these test specimens were exposed to different maximum temperatures of 250 and 350 °C. Even with exposure to high temperature, most test specimens in the third series still displayed considerable strength and strain capacity, except for the L200-250 and L200-350 specimens. At the final failure, the ultimate strains ($\varepsilon_{u}$) of the TRM specimens subjected to the maximum temperature of 250 °C were in a range from 0.0162–0.0256; meanwhile, the ultimate strain ranging from 0.0063 to 0.0098 was achieved for the TRM specimens subjected to the maximum temperature of 350 °C. However, due to the exposure to high temperature, most test specimens showed a load drop or fluctuation in the experimental curves after the cracking of the mortar matrix. Following that, the test specimens exhibited non-linear behavior and rupturing of the carbon fiber filaments. However, in the cases of the specimens exposed to a temperature level of 350 °C, the stress–strain curves showed severe non-linear behavior after mortar cracking. 

**Figure 17:**
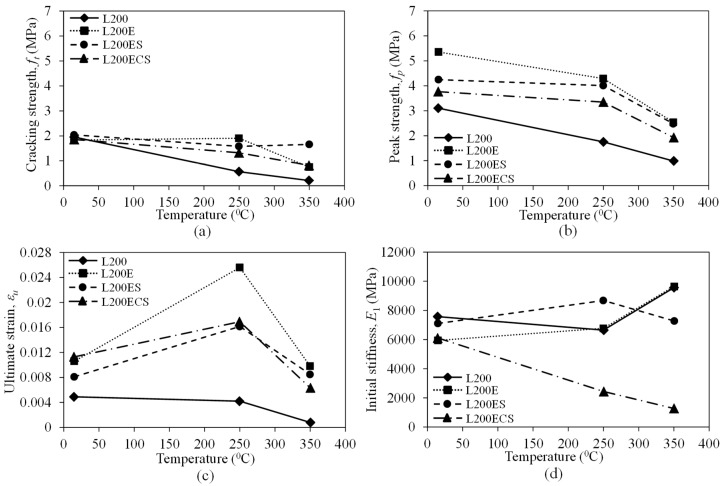
Evolution of (**a**) cracking strength, (**b**) peak strength, (**c**) ultimate strain, and (**d**) initial stiffness according to temperature of the TRM specimens in the third series.

Figure 17 presents the evolution of cracking strength, peak strength, ultimate strain, and initial stiffness of the TRM test specimens in the third series according to temperature. In Figure 17a–c, it can clearly be seen that although different details were used in the overlapping region, all test specimens in the third series exhibited nearly the same trends in cracking strength, peak strength, and ultimate strain. In Figure 17a,b, it can be seen that the cracking and peak strengths decreased as the high temperature increased. 

The same behaviors were also found in the study by Tlaiji et al. [41]. Note that in the study by Tlaiji et al. [41], continuous alkali-resistant (AR) glass textiles were used as reinforcements. The degradation of the cracking strengths of the TRM specimens exposed to the high temperature can be explained by the fact that the first stage (un-cracked stage) of the stress–strain curves is mainly dependent on the properties of the mortar matrix. Under high temperature, the tensile strength of the mortar matrix was decreased, thus leading to the crack development in the mortar matrix under low tension loading [42]. In terms of peak strength, according to Nguyen et al. [24], the reduction of the peak strength following exposure to high temperature was attributed to the alteration or degradation of the interfacial bonding between textile reinforcement and mortar matrix. The alteration or degradation of the interface might cause a reduction of the interaction between textile reinforcement and mortar matrix, and this would be magnified because of the difference in thermal expansion between carbon fibers and mortar. In reality, the mortar matrix would be contracted when subjected to high temperature, due to the evaporation of the water in the mortar mixture. The thermal contraction of mortar combined with the thermal expansion of carbon fiber textile would provide increased abundance to the stress state at the interface between textile reinforcement and mortar matrix, thus contributing to the degradation of the textile reinforcement-mortar interaction. Moreover, the degradation of the tensile strength of the carbon fiber textile exposed to high temperature partially contributed to the reductions of the peak strengths of the TRM specimens [43]. 

In Figure 17c, aside from the L200-250 specimens, the other test specimens showed significant increases of ultimate strains at a curing temperature of 250 °C. This is because after the cracking of the mortar matrix, the stress–strain curve characteristics of the TRM specimens are mainly governed by the mechanical rigidity of the carbon fiber textile reinforcement. Following exposure to the maximum temperature of 250 °C, the mechanical rigidity of carbon fiber textile was degraded, which explains the larger strains of the TRM specimens. When the applied temperature increased up to 350 °C, such strains decreased and reached almost the same values as those obtained at the room temperature condition. The same observations were also presented in the study by Nguyen et al. [24] and Tlaiji et al. [41]. These were attributed to the effect of high temperature and the mortar matrix properties as well as the fact that the interfacial bonding between mortar matrix and textile reinforcement was diminished, which could cause damage to the test specimens prior to tensile testing. Such damage was expected to limit the strain capacities of the TRM specimens [24]. 

In the case of initial stiffness, as shown in Figure 17d, aside from the L200ECS specimen, the initial stiffness remained nearly constant, even in high temperature, for the other test specimens. From Figure 17, it can be seen that the test specimens using the details in the lap splice presented higher cracking strength, peak strength, and ultimate strain than those of the control specimens in the L200 series, even though they were exposed to high temperature. This observation confirmed the effectiveness of the details used in the overlapping region, even with exposure to high temperature. In addition, as shown in Figure 17a–c, at both temperature levels of 250 and 350 °C, the specimens in the L200E series impregnated by epoxy still exhibited higher cracking strength, peak strength, and ultimate strain than the other details, except for the case of cracking strength at the temperature level of 350 °C. 

## 4. Prediction of Tensile Behaviors for TRM Composites Exposed to High Temperature

Many experimental studies were performed to investigate the behavior of the polymer composite materials after exposed to high temperature, and various analytical models were also developed to predict the material behaviors [44]. On the contrary, a few analytical models to predict the TRM materials, especially, exposed to high temperature were developed. In this study, a prediction model developed by Gibson et al. [45] for predicting the tensile behaviors of the TRM composite specimens subjected to high temperature was adopted, and verified by the experimental results.

Gibson et al. [45] proposed a general curve-fitting analysis method through the hyperbolic tangent functions to fit test data considering the effect of temperature on material behaviors.

(2) $$P\left(T\right)=\left\{\frac{P_{0}+P_{u}}{2}-\frac{P_{0}-P_{u}}{2}\tanh\left[\zeta \left(T-T_{g}\right)\right]\right\}R^{n}$$

where P(T) is the mechanical properties at temperature T, $P_{0}$ is the mechanical properties at room temperature (approximately 11 °C), and $P_{u}$ is the mechanical properties at high temperature obtained by fitting the experimental data. In this study, the values of $P_{u}$ were determined at 350 °C; except for L200E in term of cracking strength and L200ES in term of initial stiffness, of which $P_{u}$ was determined as a function of temperature as shown in Table 3. ζ is the coefficient considering the effect of temperature and was determined by fitting the experimental data. In this study, for 11 °C≤T≤350 °C, a constant value of ζ (=0.014) was used, which was the same as that given in Homoro et al. [46]. Note that in the study by Homoro et al. [46], such coefficient was applied to fit the ultimate strength of the TRM specimens subjected to the temperature ranging from 150 °C to 600 °C. $T_{g}$ is the glass transition temperature, and is determined to be the point where the peak strength-temperature curves (or cracking strength-temperature and initial stiffness-temperature curves) are almost symmetrical [46]. R (=0.958) is the volume fraction of the mortar matrix considering the decomposition after exposed to high temperature. In this study, it is assumed that the change of mortar matrix content due to the temperature effect was not considerable, thus R is constant and the same for all test specimens. Finally, n is a material parameter affecting the mechanical properties of the TRM composites (n=0 for carbon fiber textile and n=1 for mortar matrix [46]).

The calibrated parameters for TRM composites are presented in Table 3. Because the model developed by Gibson et al. [45] was in a general form, thus it would be used to predict not only the peak strength ($f_{p}$) but also the cracking strength ($f_{t}$) and initial stiffness ($E_{1}$) of the test specimens. 

**Figure 18:**
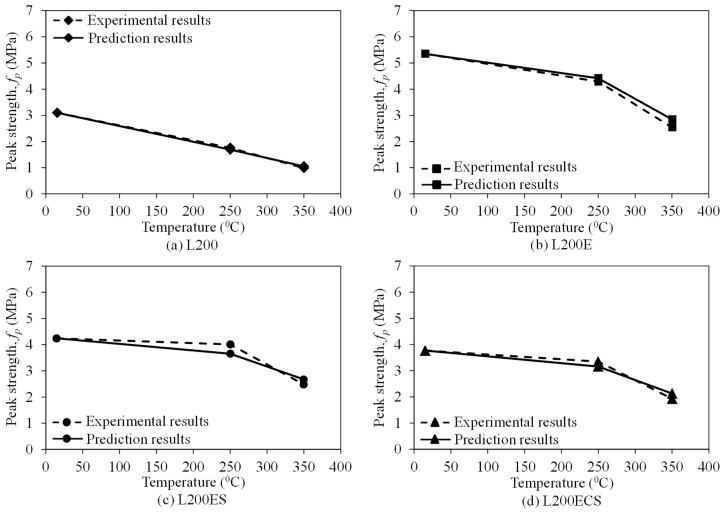
Predicted peak strengths of the TRM specimens according to temperature.

**Figure 19:**
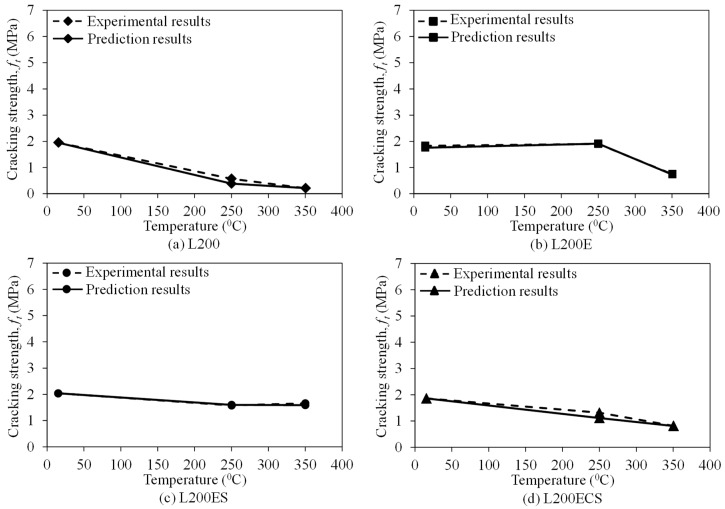
Predicted cracking strengths of the TRM specimens according to temperature.

**Figure 20:**
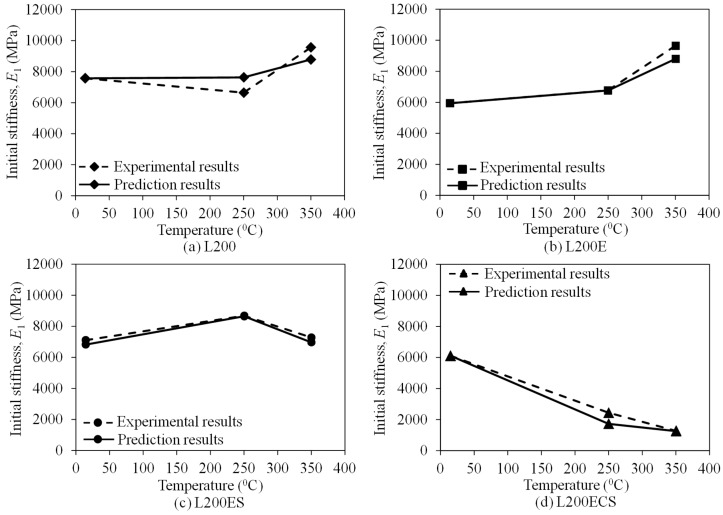
Predicted initial stiffness of the TRM specimens according to temperature.

Figure 18, Figure 19 and Figure 20 compare the peak strength, cracking strength, and initial stiffness according to temperature acquired from the test results and prediction results by the Gibson model (Equation (2)). In general, the peak strength, cracking strength, and initial stiffness curves predicted by the Gibson model show a good agreement with the experimental ones. In addition, as presented in Table 3, the ratio between the peak strength obtained from the test results and predicted by the model was in a narrow range of 0.89–1.10, that of cracking strength was from 0.98 to 1.49, and that was 0.87–1.41 for initial stiffness. This indicates that the model developed by Gibson et al. [45] could be used to predict the tensile behaviors of the lap-spliced carbon fiber-textile reinforced mortar composites exposed to high temperature with a reliable accuracy. 

## 5. Conclusions

TRM specimens comprised of lap-spliced carbon fiber-textile reinforcement and aluminum cement-based mortar were tested in this study in order to investigate their tensile behaviors by employing direct tensile tests. Three different details in the lap splice region including impregnating carbon fiber textile with epoxy, aluminum oxide powder, and covering with one layer of carbon fiber fabric were developed in attempts to enhance the mechanical properties of TRM specimens. Three different lap splice lengths of 180, 200, and 220 mm were also used. The effect of high temperature on the tensile behaviors of TRM specimens were also investigated through two different temperature histories with maximum temperatures of 250 °C and 350 °C, respectively. From the test results, the primary findings are as follows:

1. The failure modes of the TRM test specimens in this study were essentially governed by carbon fiber rupturing after cracking occurred in the mortar matrix. In addition, the high temperature did not affect the failure modes of the test specimens despite causing strength reductions.
2. In the case of the control specimens without surface treatment, after the cracking of the mortar matrix, the test specimens showed linear stress–strain curves up to the peak load, then a sudden load drop was observed. In contrast, the other test specimens with surface treatment in the overlapping region and/or exposure to high temperature showed non-linear behavior with fluctuation and gradual failure. However, despite showing fluctuation, the specimens with surface treatment in the overlapping region and/or exposure to high temperature displayed approximately trilinear behavior with un-cracked and cracked stages that were similar to the control specimens.
3. The use of various lap splice lengths did not change the initial stiffness ($E_{1}$) or cracking strength ($f_{t}$), but increased the peak strengths ($f_{p}$) of the TRM specimens. In addition, the elastic moduli of cracked stage ($E_{2}$) and ultimate strain ($\varepsilon_{u}$) fluctuated with different lap splice lengths.
4. The surface treatment in the overlapping region did not significantly change the initial stiffness, cracking strength, or elastic modulus of the cracked stage of the TRM specimens, but significantly increased the peak strength and ultimate strain compared to those of the non-detailed specimen L200-RT.
5. The same trends in cracking strength, peak strength, and ultimate strain were observed for the TRM test specimens exposed to high temperature. The cracking and peak strengths decreased as the temperature increased. The ultimate strains of the test specimens using the details in the overlapping region were substantially higher than those of the test specimens in the L200 series, despite exposure to high temperature. Under high temperature, most test specimens showed nearly constant initial stiffness, except for the L200ECS specimens.
6. The prediction model developed by Gibson et al. [45] was adopted to predict the tensile behaviors including peak strength, cracking strength, and initial stiffness of the TRM composites. The predicted results indicated that the Gibson model could be used to evaluate the tensile behaviors of the lap-spliced carbon fiber-textile reinforced mortar composites exposed to high temperature with a good agreement with the experimental results.

Finally, it is noted that the conclusions mentioned above were based on limited test data in this study. In particularly, more test specimens exposed to high temperature could be beneficial to the improvement of the results’ reliability. Also, a wide range of lap splice lengths, effect of mortar types, and specimen configurations need to be examined for long-term performance of concrete members after strengthening.

## Figures and Tables

**Table 1:** Properties of TRM specimens in this study.

Specimens	Total Length (mm)	Lap Splice Length or Central Length (mm)	Gripping Length (mm)	Coating Method	Exposure to Fire	Number of Test Specimens
L180-RT	450	180	125	- ^(1)^	- ^(1)^	2
L200-RT	450	200	115	- ^(1)^	- ^(1)^	2
L220-RT	450	220	105	- ^(1)^	- ^(1)^	2
L200E-RT	450	200	115	Epoxy	- ^(1)^	3
L200ES-RT	450	200	115	Epoxy + sand blasting (aluminum oxide powder)	- ^(1)^	3
L200ECS-RT	450	200	115	Epoxy + carbon fiber fabric + sand blasting (aluminum oxide powder)	- ^(1)^	3
L200-250	450	200	115	- ^(1)^	Max. temp.: 250 °C	2
L200-350	450	200	115	- ^(1)^	Max. temp.: 350 °C	1
L200E-250	450	200	115	Epoxy	Max. temp.: 250 °C	2
L200E-350	450	200	115	Epoxy	Max. temp.: 350 °C	1
L200ES-250	450	200	115	Epoxy + sand blasting (aluminum oxide powder)	Max. temp.: 250 °C	2
L200ES-350	450	200	115	Epoxy + sand blasting (aluminum oxide powder)	Max. temp.: 350 °C	1
L200ECS-250	450	200	115	Epoxy + carbon fiber fabric + sand blasting (aluminum oxide powder)	Max. temp.: 250 °C	2
L200ECS-350	450	200	115	Epoxy + carbon fiber fabric + sand blasting (aluminum oxide powder)	Max. temp.: 350 °C	1

^(1)^ Not applicable.

**Table 2:** Tensile test results of TRM specimens in this study.

Specimens	Initial Stiffness		Cracking Strength		Cracking Strain		Elastic Modulus of Cracked Stage		Peak Strength		Ultimate Strain		Failure Mode
	*E_1_* (MPa)	COV	*f_t_* (MPa)	COV	*ε_t_*	COV	*E_2_* (MPa)	COV	*f_p_* (MPa)	COV	*ε_u_*	COV	
L180-RT	7770.8	0.102	2.12	0.026	0.00028	0.129	502.3	0.197	3.03	0.134	0.0023	0.271	A
L200-RT	7573.0	0.229	1.95	0.036	0.00026	0.194	318.3	0.180	3.10	0.021	0.0049	0.216	A
L220-RT	8983.4	0.208	2.00	0.002	0.00023	0.207	544.0	0.123	3.42	0.041	0.0028	0.182	A or B
L200E-RT	5941.8	0.280	1.83	0.177	0.00032	0.127	350.9	0.128	5.35	0.137	0.0106	0.239	A or B
L200ES-RT	7106.5	0.087	2.04	0.030	0.00029	0.062	355.7	0.168	4.24	0.172	0.0081	0.212	A or B
L200ECS-RT	6104.4	0.410	1.86	0.219	0.00033	0.306	308.0	0.653	3.77	0.101	0.0090	0.545	A or B
L200-250	6644.2	0.084	0.57	0.145	0.00009	0.228	-	-	1.76	0.084	0.0042	0.660	A
L200-350	9562.5	-	0.21	-	0.00002	-	-	-	0.99	-	0.0008	-	A
L200E-250	6769.4	0.335	1.90	0.071	0.00029	0.267	-	-	4.29	0.274	0.0256	0.232	A or B
L200E-350	9642.9	-	0.75	-	0.00008	-	-	-	2.54	-	0.0098	-	A
L200ES-250	8679.8	0.537	1.58	0.087	0.00019	0.184	-	-	4.01	0.091	0.0162	0.473	A or B
L200ES-350	7280.5	-	1.66	-	0.00023	-	-	-	2.48	-	0.0085	-	A
L200ECS-250	2440.9	0.533	1.32	0.335	0.00069	0.797	-	-	3.35	0.009	0.0169	0.181	A or B
L200ECS-350	1278.3	-	0.82	-	0.00064	-	-	-	1.93	-	0.0063	-	B

**Table 3:** Calibrated parameters used in prediction model.

Specimens	$P_{0}$	$P_{u}$	ζ	$T_{1}\;({}^{\circ}C)$	$T_{2}\;({}^{\circ}C)$	$T_{g}\;({}^{\circ}C)$	$P\left(T_{1}\right)$	$P\left(T_{2}\right)$	$f_{p}/P\left(T_{1}\right)$ ^(1)^	$f_{p}/P\left(T_{2}\right)$ ^(1)^
(a) Peak strength (MPa)										
L200	3.10	0.99	0.014	250	350	225	1.69	1.05	1.04	0.94
L200E	5.35	2.54	0.014	250	350	275	4.42	2.85	0.97	0.89
L200ES	4.24	2.48	0.014	250	350	275	3.66	2.67	1.10	0.93
L200ECS	3.77	1.93	0.014	250	350	275	3.16	2.13	1.06	0.91
(b) Cracking strength (MPa)										
L200	1.95	0.21	0.014	250	350	175	0.38	0.21	1.49	0.98
L200E	1.83	0.62 ^(2)^	0.014	250	350	280	1.91	0.74	0.99	1.02
L200ES	2.04	1.66	0.014	250	350	125	1.60	1.59	0.99	1.04
L200ECS	1.86	0.82	0.014	250	350	225	1.12	0.81	1.18	1.01
(c) Initial stiffness (MPa)										
L200	7573.0	9562.5	0.014	250	350	300	7632.0	8783.8	0.87	1.09
L200E	5941.8	9642.9	0.014	250	350	280	6761.4	8800.1	1.00	1.10
L200ES	7106.5	6965.2 ^(3)^	0.014	250	350	250	8645.5	6965.2	1.00	1.04
L200ECS	6104.4	1278.3	0.014	250	350	175	1729.0	1258.8	1.41	1.02

^(1)^$f_{t}/P\left(T_{1}\right)$ and $f_{t}/P\left(T_{2}\right)$ for cracking strength, and $E_{1}/P\left(T_{1}\right)$ and $E_{1}/P\left(T_{2}\right)$ for initial stiffness; ^(2)^ Predicted value by $P_{u}\left(T\right)=6.78-0.0176\times T$ for 11 °C≤T≤350 °C; ^(3)^ Predicted value by $P_{u}\left(T\right)=20097.5-36.62\times T$ for 11 °C≤T≤350 °C.
